# Platelet-Rich Fibrin Promotes an Accelerated Healing of Achilles Tendon When Compared to Platelet-Rich Plasma in Rat

**Published:** 2015-07

**Authors:** Franciele Dietrich, Gustavo L. Duré, Caroline P. Klein, Vinícius F. Bampi, Alexandre V. Padoin, Vinícius D. Silva, Jefferson Braga-Silva

**Affiliations:** 1 Laboratory of Medical Abilities and Surgical Research, Pontifícia Universidade Católica do Rio Grande do Sul (PUCRS), Porto Alegre, Rio Grande do Sul, Brazil;; 2 Service of Hand Surgery and Reconstructive Microsurgery, São Lucas Hospital, Pontifícia Universidade Católica do Rio Grande do Sul (PUCRS), Brazil;; 3 Service of Pathology Anatomy and Cytopathology, São Lucas Hospital, Pontifícia Universidade Católica do Rio Grande do Sul (PUCRS), Brazil

**Keywords:** Healing, Platelet-rich fibrin, Achilles tendon, Platelet-rich plasma

## Abstract

**BACKGROUND:**

Autologous platelet concentrate has been used to improve the function and regeneration of injured tissues. Tendinopathies are common in clinical practice, although long-term treatment is required. On the basis of lead time, we compared the effect of using platelet-rich plasma (PRP) and platelet-rich fibrin (PRF) in repairing rat Achilles tendon.

**METHODS:**

The effectiveness of using PRP and PRF was evaluated after 14 and 28 postoperative days by histological analysis. The quantification of collagen types I and III was performed by Sirius red staining. Qualitatively, the data were verified with hematoxylin-eosin (H&E) staining.

**RESULTS:**

In Sirius red staining, no significant treatment differences were found between groups. Statistical difference was observed only between PRP (37.2% collagen) and the control group (16.2%) 14 days after treatment. Intra-groups compared twice showed a difference for collagen I (27.8% and 47.7%) and III (66.9% and 46.0%) in the PRF group. The control group showed differences only in collagen I (14.2% and 40.9%) and no other finding was observed in the PRP group. In H&E staining, PRF showed a better cellular organization when compared to the other groups at 28 days.

**CONCLUSION:**

Our study suggests that PRF promotes accelerated regeneration of the Achilles tendon in rats, offering promising prospects for future clinical use.

## INTRODUCTION

Tendon injuries are a challenge in orthopedic medicine, with an annual incidence estimated at one break per 10,000 people,^1^ affecting the whole population and athletes.^2^ Total rupture or accidental tendon transects, generally indicate surgery and ideal surgical approach is to bring the two stumps, accelerating the healing,^3^ however a long time of immobilization^4^ and recovery^5^ is required. This scenario has prompted the development of research aimed at the improvement and acceleration of the repair and regeneration of injured tendons, and tissue engineering has given rise to a way to accelerate the repair process^6^ for tendons and other tissues, attributing to platelets a promising role, since they play a key role in homeostasis.^7^

Platelet-rich plasma (PRP) is a plasma volume with a high platelet count,^8^ and it is known to have high concentrations of different growth factors,^9^^,^^10^ Therefore, it is seen as a natural source of growth factors, with easy acquisition and low cost.^11^ Several studies have demonstrated the efficacy of PRP in the repair and regeneration of various tissues^12^^-^^15^ including tendons.^16^^-^^18^ In contrast, other studies have not shown the clinical superiority of PRP in the repair or regeneration tissues such as nerves^19^ and tendons.^20^^,^^21^

Platelet-rich fibrin (PRF) consists of a concentrate of autologous platelets on a fibrin membrane without added external factors^   22^ and has a high potential for tissue repair.^23^^,^^24^ In angiogenesis, PRF acts in stimulation phase, then occurring vascular growth and increased collagen synthesis throughout fibroblast proliferation.^   25^ So, its use has been proposed as a strategy to enhance the cellular response to tendon injury and the quality of repair.^26^ The aim of this study was to compare the effect of PRP and PRF in repair of rat Achilles tendon injuries.

## MATERIALS AND METHODS

*Animal Model*

Fifty-four adult male Wistar rats, weighing approximately 280 g, were used in this study. Achilles tendon (AT) was transected in forty-eight rats, and the six remaining animals were used as blood donors to obtain PRP and PRF. All the procedures carried out and listed bellow were previously approved by the Ethics Committee of the Pontifical Catholic University of Rio Grande do Sul (no. 10/00191 CEUA-PUCRS) and all efforts were made to minimize animal suffering and to reduce the number of animals used. The animals were maintained in ventilated racks under controlled temperature (22˚C) and with a 12 h light/dark cycle.

The rats were anesthetized with ketamine hydrochloride (Dopamin®, Cristália, São Paulo, Brazil 60 mg.kg^-1^ of animal weight) and meperidine (Dornot®, União Química, São Paulo, Brazil 20 mg.kg^-1^) both given intramuscularly (IM) in the left hind leg. The right hind leg was shaven, and the AT was then exposed. Using a surgical microscope (Zeiss, Oberkochen, Germany), the AT was cut transversely in the middle portion, and the two severed ends were ligated with a Kessler-type suture,^27^ using mononylon 7-0 (Ethicon® Somerville, Johnson and Johnson, São Paulo, Brazil). 

At the lesion site, immediately following suturing, 50 μL^28^ of the treatments related to each experimental group (n=16) were applied: PRP, PRF and control (saline). The skin was sutured, using Vicryl 5-0 (Ethicon®, Johnson and Johnson, São Paulo, Brazil). Ketoprofen (Cetoprofen®, Eurofarma Laboratórios, São Paulo, Brazil 5 mg/kg, I.M.) was administered subcutaneously once daily for 3 days as analgesic and anti-inflammatory. The assessments were performed at 14 (n=8) and 28 days (n=8) for each group, following euthanasia performed by intra-cardiac injection with a lethal dose of thiopental (Thiopental, Cristália, São Paulo, Brazil). The AT injured area was removed for histological evaluation. 

*Platelet-Rich Plasma Preparation*

Blood was collected into a syringe containing acid citrate dextrose anticoagulant (ACD Solution A-Becton Dickinson, NJ, USA) from donor animals via cardiac puncture. The blood sample was centrifuged (Centrifuge Centribio-80-2B, Rio de Janeiro, Brazil) twice^29^ at 250 *g* for 10 minutes and then 1000 *g* for 10 minutes. The platelets were obtained in high concentration (600 µL, PRP). A 50-µL portion of PRP was activated with calcium chloride (Sigma-Aldrich, St. Louis, MO, USA) plus thrombin and applied to each lesion. The platelet count was determined with an automatic blood counter (Vet Abc Plus+Horiba Medical, Gurnee, IL, USA).

*Platelet-Rich Fibrin Preparation*

The blood used to obtain the PRF was collected without addition of anticoagulant and was submitted to one centrifugation, at 400 *g* for 10 minutes.^24^ The PRF membrane was immediately withdrawn from the tube and separated from the remaining blood. A platelet count was obtained and a 50-µL volume of PRF was applied to the lesion.

*Histological Analysis*

The tissues removed were fixed in 10% buffered formalin for 24 hours, dehydrated, cleared in xylene and embedded in paraffin. Two longitudinal sections (4 µm) were made from the peripheral and central areas of each animal. The depth of the cut was established after the block was sectioned 15 times. It was selected 9 representative fields from each section in hematoxylin-eosin (HE) and Sirus red staining (SRS). Three blind observes examined each histologic parameter independently.

SRS was visualized under polarized light microscopy (Zeiss Axioskop 40 optical Cool SNPAP^TM ^Pro cf, Göttingen, Germany) and used to quantify the different types of collagen present in the AT: yellowish-red color associated with thick type I collagen fibers and greenish color with thin type III collagen fibers.^30^ Pixel counts were obtained from the three selected fields for each animal (both central and peripheral) and converted to percentages. All histological images were analyzed with Image Pro Plus® 4.5.1 software (Media Cybernetics, Inc., Rockville, MD, USA). HE was used to determine qualitatively vascular proliferation, mononuclear and polymorphonuclear cells, and fibroblastic and epithelial cells, which were visualized by light microscopy.

*Statistical Analysis*

The quantitative results were expressed as mean±standard error (SE), and analyzed by one-way ANOVA and two-way repeated measures followed by Bonferroni *post hoc *test, using the software Graph Pad Prism (La Jolla, CA, USA). The significance level was set at *p*<0.05.

## RESULTS

*Platelet Count*

The platelet count in whole blood ranged from 286,000 to 502,000/µL. The platelet count in the PRP group increased up to 12 times (2,616,000 to 4,080,000/µL) corresponding to increased platelet aggregation (676 to 1136%). The leukocyte count also increased in PRP group (36 to 125%). In the PRF, the platelet count decreased, ranging from 14,000 to 55,000/µL. 

*Sirius Red Staining*

**Fig. 1:**
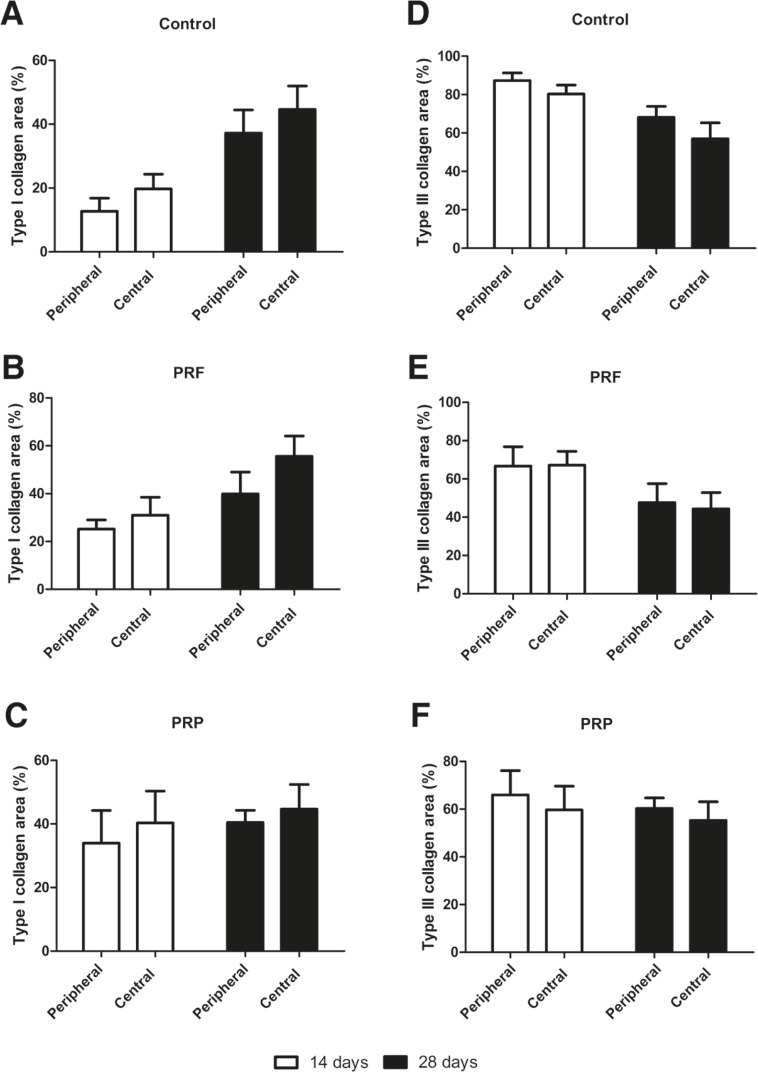
Central and peripheral analysis of type I collagen (A, B, C) and type III collagen (D, E, F) at 14 and 28 days (n=8).

**Fig. 2:**
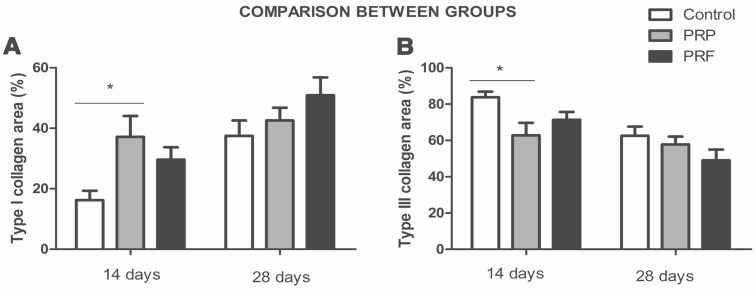
Comparison between type I and III collagen areas in different groups at 14 and 28 days. * p <0.05 (n=8).

The intra-group analysis for the two types of collagens showed no significant difference with regard to the central and peripheral cuts, at either evaluation time (*p*>0.05) (Figure 1). Thus, central and peripheral cuts were joined to analysis. In relation to type I collagen area, post-hoc analysis identified a statistical difference only between the control and PRP groups at 14 days after treatment (*p*=0.01). Comparisons at 28 days indicated no statistically significant difference between all groups (control, PRP and PRF) (*p*>0.05) (Figure 2).

The same analysis was performed for type III collagen and showed statistical difference between the PRP and control groups (*p*=0.034) at 14 days. However, there was no significant difference between the control and PRP groups at 28 days. At both times, there was no significant difference between the control and PRF groups or between the PRF and PRP groups (*p*>0.05) (Figure 2).

**Fig. 3:**
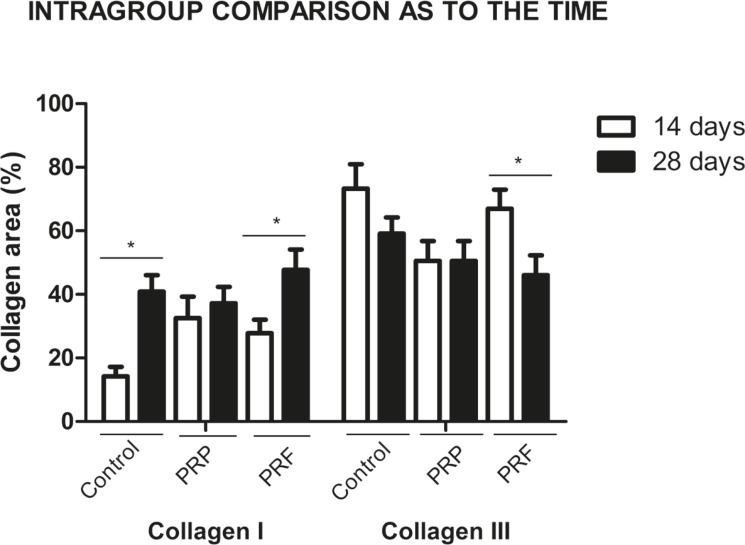
Intragroup comparison as to time and area of type I and III collagen. * p<0.05 (n=8)**.**

**Fig. 4:**
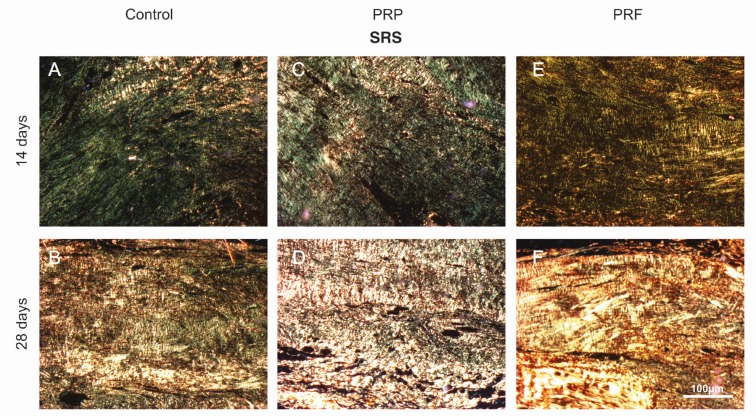
Images captured with Sirius red staining (SRS) in a longitudinal section after treatments, at 14 (A, C, E) and 28 (B, D, F) days. Magnification: X50 Bar: 100 µm

Comparing type I collagen areas at the two times (14 and 28 days), a statistical difference was noticed in the control (*p*=0.01) and PRF (*p*<0.05) groups. The PRP group remained stable for the collagen type I and III indices over time. However, only the PRF group showed a statistical difference both to type I as to type III collagen indices between the two evaluated time periods (*p*<0.05) (Figure 3), and this can also be checked in the SRS images (Figure 4). Ckecking this information, there was a significant type I collagen increase and a type III collagen decrease in PRF group over time, suggesting a better healing process.

*Hematoxylin-Eosin Staining*

**Fig. 5:**
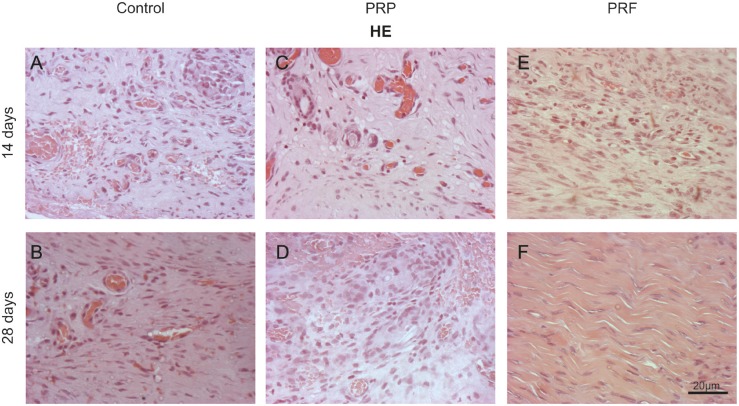
Images captured with Hematoxylin-eosin (HE) in a longitudinal section after treatments, at 14 (A, C, E) and 28 (B, D, F) days. Magnification: X200 Bar: 20 µm

Hematoxylin-eosin staining confirmed the quantitative data previously obtained (Figure 5).

Histological analysis showed no difference between the groups at either time (14 or 28 days) with regard to the central and peripheral cuts. Qualitatively, the cellularity level showed to be similar in the PRP and control groups at 14 days. The PRP group displayed an active granulation tissue and large hemorrhagic area. However, the PRF compared to the control group showed an advanced process of neovascularization and a reduced inflammatory infiltrate, without the presence of granuloma. A qualitative difference was observed between the PRP and PRF groups: the first group showed tissue disorganization, an abundant inflammatory infiltrate, hemorrhagic area and mononuclear and polymorphonuclear cells, while the formation of new vessels was more prevalent in the latter.

At 28 days, despite the hemorrhagic area in the PRP group, comparison with the control group showed microscopic similarities: an enhanced cellularity with a substantial amount of young fibroblasts, less collagen fibers, fibrocytes and fibrosis. On comparing the three groups, a qualitative difference was observed in the PRF group, as it showed a more organized tissue, a large number of fibrocytes and a low amount of fibroblasts, characterizing a partially healed tissue. On the basis of the presence of different cell types, the number of blood vessels and the arrangement of collagen fibers at 14 and 28 days, it can be indicated that there was a more progressive tissue repair in the PRF and control groups than in the PRP group (Figure 5).

## DISCUSSION

There have been published studies aimed at improving tissue repair in tendon injuries, since tendon has a low capacity for regeneration due low oxygenation and nutrition.^30^ It is known that tendon repair is not controlled by a single growth factor, but rather it requires the interaction of various factors.^31^ The true effectiveness is dependent on their concentration,^32^ prompting the evaluation of two different platelet concentrates in order to obtain a suitable concentrate for a quicker tendon repair and regeneration. However, to the best of our knowledge, there are no studies available in the current literature that evaluated and compared the effect of PRP and PRF on tendon regeneration. 

Marx and coworkers (1998)^33^ proposed that PRP must have an increased platelet count of more than 338%, with basal concentrations of 300,000 platelets/µL,^34^ which is considered an ideal number for treatment with beneficial effects.^35^ In our study, the platelet count average in PRP reached 906.26% compared to the average basal count. At low concentrations (<1,000,000 platelets/µL), PRP has less notable effects, and paradoxically, at high concentrations (>1,000,000 platelets/µL), it seems to exert an inhibitory effect.^32^ According to Marx (2001),^8^ the concentration of platelets used as reference for the therapeutic use of PRP is 1,000,000 platelets/µl; we used this platelet count in our study.

Even though there are studies showing enhanced regeneration and repair in tendinopathy,^16^ the true effectiveness of PRP is still controversial.^33^ Our study showed that the use of PRP, when applied immediately after the lesion, is not effective in accelerating the regeneration process of the AT in rats. Unlike PRP, PRF (a natural part of fibrin) is prepared usually without the addition of thrombin, which may protect growth factors from proteolysis.^36^ Therefore, it is possible to suggest that PRF had a continuous action, noted be a presenting no significant improves in AT repair after 14 days. 

The PRF production protocol is an attempt to accumulate platelets and release cytokines in a clot fibrin^23^ so the basal count must be diminished. Our data support these precepts, showing a suppression of platelets in the fibrin clot ranging from 89.04 to 95.10% of the theoretical basal concentrations in whole blood. Growth factors present in the fibrin clot are incorporated during the process of polymerization within the platelets and fibrin.^23^ This factors have controlled effects, since they are gradually released over the time.^37^

The number of fibroblasts and the proliferation of new vessels decreases, while the extracellular matrix deposition increases in the healing process.^38^ Hence, it was possible to notice that after 28 days, only the PRF group (Figure 1) exhibited a vascular regression. At 14 days, the lesion site was highly vascularized. According to Abbas (2008),^38^ growth factors stimulate the proliferation of some cells and inhibit others, and thus, depending on their concentrations, they can have opposite effects in the same cell. These concentrations were not measured in this study, so this missing information could have explained the inefficacy of PRP. The growth factors involved in tissue repair are synthesized by leukocytes recruited and activated at the injury site, as part of the inflammatory process.^38^ Prior to PRP treatment, the blood cell counts showed an increase of 33 to 125% in leukocytes in relation to whole blood, suggests the presence of growth factors within PRP prepared by our group, excluding the possibility of a lack of effect of PRP on tendon a more organized tissue, a more organized tissue repair.^38^

Autologous PRF has been proposed to improve the cellular response to tendon injury as well as the quality of repair,^25^ corroborating with the analyses of our study. We demonstrated that the PRP and the control group showed similar effects in rat AT regeneration at 28 days in relation to collagen type I and III areas (Figure 2 and 4), while the PRF treatment accelerated the healing process since the type I collagen area was greater than in the other groups at 28 days, although without significance (Figure 2).

The PRF treatment showed a delay in the peak of growth factor release of over 14 days, compared to PRP. PRF showed a release peak after 14 days, where levels remained higher than in the PRP group,^38^ explaining the stagnation of the appearance of type I collagen at 28 days in the PRP group. Thus, the data demonstrated that the PRF group had a tendency toward enhanced healing, since the type I collagen area was greater at 28 days when compared to the PRP and the control groups, although without significance. The PRP group at 14 days demonstrated a greater type I collagen area and smaller type III collagen area, significantly different compared to the control group (Figure 2). However, at 28 days, the type III collagen area remained the same as at 14 days in the PRP group (Figure 3), explained by the massive and uncontrollable effect in the short term, in which the release of growth factors peaks on the first day, resulting in rapid collagen polymerization because of the high thrombin levels.^23^

On the basis of microscopic analysis with H&E (Figure 5), the PRF group demonstrated better healing at both assessment times, compared to the PRP and the control groups, where the latter groups showed no difference. In addition, hemorrhagic areas were noted in the PRP group at both times and in the control group at only 14 days. 

Type I and III collagen are the main components of the extracellular matrix in tendons, corresponding to 65-95% and 10% of the composition, respectively. The synthesis of type III collagen increases during the early repair stages, and it is believed that when it decreases, type I collagen is synthesized and organized.^39^ The intra-group analysis with SRS (Figure 3), at 14 and 28 days, showed a significant difference in the PRF group between type I and III collagen areas (*p*<0.05), similar to the normal tendon healing. The same occurred with type I collagen in the control group (*p*<0.05). On the contrary, type I and III collagen areas in the PRP group at 14 and 28 days did not differ significantly, explaining the delayed healing. Our findings allowed us to conclude that PRF has a trend towards improvement and acceleration of healing when compared to PRP, in an animal model of AT repair. It is possible to suggest the use of PRF may be a promising alternative in orthopedic surgery, although further experiments are required to support our results.

## Note

Please cite this paper as:

Dietrich F, Duré GL, Klein CP, Bampi VF, Padoin AV, Silva VD, Braga-Silva J. Platelet-Rich Fibrin Promotes an Accelerated Healing of Achilles Tendon When Compared to Platelet-Rich Plasma in Rat. World J Plast Surg 2015;4(2):101-109.
